# Introduction to *Mammalian Genome* Special Issue: Epigenetics

**DOI:** 10.1007/s00335-020-09843-3

**Published:** 2020-07-08

**Authors:** Johannes Beckers, Raffaele Teperino, Yann Hérault, Martin Hrabé de Angelis

**Affiliations:** 1 Institute of Experimental Genetics, Helmholtz Zentrum München GmbH, Neuherberg, Germany; 2 Department of Molecular Life Sciences, Chair of Experimental Genetics, Technical University Munich, Freising, Germany; 3 DZD – German Center for Diabetes Research, Neuherberg, Germany; 4 Université de Strasbourg, CNRS UM7104, INSERM U1258, IGBMC, PHENOMIN-ICS, Illkirch, France

*Mammalian Genome* published its last Special Issue on the topic of epigenetics in 2009. Now, 11 years later we have collected reviews and original papers for a second issue on epigenetics. It is thus a good time to look at how the field of epigenetics has progressed within this last decade. The articles in the 2009 Special Issue mostly covered the topics of genomic imprinting, X-chromosome inactivation and the role of epigenetics in human diseases such as cancer, deafness, psychiatric disorders, and diabetes (Peters 2009). The molecular mechanisms included DNA methylation of CpGs (Glass et al. 2009; Irizarry et al. 2009), modifications of histone proteins as well as the involvement of long non-coding RNAs in imprinting clusters and X chromosome inactivation (Nagano and Fraser 2009) and miRNAS in cancer and deafness (Friedman and Avraham 2009; Valeri et al. 2009). These are probably still the most commonly studied molecular mechanisms in epigenetics, together with the more recently described role of fragmented and chemically modified non-coding RNAs. A new thematic focus in the current issue is the finding that epigenetic mechanisms also have a role in the inheritance of acquired traits across generations.

Kaspar et al. in this issue screened the current scientific literature on the topic of parental nutrition and diets and its effect on metabolic phenotypes in the offspring (Kaspar et al. 2020, vol. 31, pp. 120–134). Whereas there is convincing data from epidemiological studies and experimental proof from rodent models for intergenerational inheritance, the comprehensive inspection of the published data rather suggests that the evidence supporting transgenerational inheritance of acquired metabolic traits in mammals until now is less convincing. In utero effects, postnatal nutrition during lactation, learned behavior and other confounding factors are sometimes overlooked in publications that claim transgenerational inheritance of metabolic phenotypes following grand-parental diets. How DNA methylation and the expression of miRNAs are affected by diets, exercise and metabolic surgery in different organs and how epigenetic changes are associated with obesity in humans and rodents is reviewed by Ouni and Schürmann (2020, vol. 31, pp. 135–146). They also point towards unresolved issues such as the reversibility and the causality of epigenetic changes in obese people. In addition to environmental factors like nutrition and stress, also genetic alterations in the parental generation can affect phenotypes of wildtype offspring. Tomar and Teperino in this issue review such examples in mammals and discuss the current data in support of this type of indirect genetic inheritance (Tomar and Teperino 2020, vol. 31, pp. 147–157).

The mechanisms that are responsible for epigenetic inheritance via gametes across generations are currently being dissected. For example, it was demonstrated that small RNAs isolated from sperm of challenged fathers and subsequently injected into naïve zygotes can reproduce epigenetically inherited phenotypes. Thus, small RNAs likely represent part of the epigenetic information. To better discern universal from specific epigenetic mechanisms, it will be required to rely on a broader spectrum of mammalian models for epigenetic inheritance across generations. To this end the research article by Weyrich et al. in this Special Issue presents original data on the epigenetic inheritance of DNA methylation signatures across generations in wild guinea pigs following paternal heat exposure (Weyrich et al. 2020, vol. 31, pp. 158–170).

In addition to establishing novel animal models, progress in our understanding of epigenetics also relies on technological developments. Advances in the sensitivity and selectivity of molecular methods to detect epigenetic changes, in the automation of handling single cells as well as in in silico analysis tools recently enabled single cell genomics and epigenomics and these developments have sustained our appreciation of cellular heterogeneity. Kamies and Martinez-Jimenez in this Special Issue review how these novel single-cell ‘omics technologies are changing our understanding of common diseases like cancer, diabetes and age-related diseases, and they discuss how these technologies may impact on targeted and individualized therapies in the future (Kamies and Martinez-Jimenez 2020, vol. 31, pp. 171–181). Taken together, there is now accumulating proof that also in mammals, epigenetic signatures are at least part of the system that enables the transmission of information related to acquired phenotypes to the next generation. In addition, epigenetic mechanisms can also change (or maintain) the nucleotide sequence of DNA and the stability of the genome. In this issue Feng and Riddle provide a comprehensive overview on how epigenetic mechanisms affect genome stability by regulating centromere and telomere function, by reducing the activity of transposable elements and by controlling DNA repair pathways (Feng and Riddle 2020, vol. 31, pp. 182–196).

We sincerely hope that readers will appreciate the information that we have collected in selected areas of the growing and topical field of epigenetics. Last but not least, we wish to thank all the authors for their contributions to this Special Issue of Mammalian Genome and Louise Tinsley for her highly efficient and professional management of authors and guest editors.
